# Draft Genome Sequences of Semiconstitutive Red, Dry, and Rough Biofilm-Forming Commensal and Uropathogenic *Escherichia coli* Isolates

**DOI:** 10.1128/genomeA.01249-16

**Published:** 2017-01-26

**Authors:** Annika Cimdins, Petra Lüthje, Fengyang Li, Irfan Ahmad, Annelie Brauner, Ute Römling

**Affiliations:** a Department of Microbiology, Tumor and Cell Biology, Karolinska Institutet, Stockholm, Sweden; b Division of Clinical Microbiology, Karolinska University Hospital, Stockholm, Sweden

## Abstract

Strains of *Escherichia coli* exhibit diverse biofilm formation capabilities. *E. coli* K-12 expresses the red, dry, and rough (rdar) morphotype below 30°C, whereas clinical isolates frequently display the rdar morphotype semiconstitutively. We sequenced the genomes of eight *E. coli* strains to subsequently investigate the molecular basis of semiconstitutive rdar morphotype expression.

## GENOME ANNOUNCEMENT

Biofilms are multicellular microbial communities surrounded by a self-produced extracellular matrix adherent to each other, to interfaces, and/or to biotic or abiotic surfaces (1). The red, dry, and rough (rdar) biofilm morphotype defined by the expression of amyloid curli fibers and the exopolysaccharide cellulose is expressed by several species of *Enterobacteriaceae*, among them *Escherichia coli* (2). Formation of the rdar morphotype is activated by the orphan transcriptional regulator CsgD and occurs predominantly at temperatures below 30°C in model strains of *E. coli* and *Salmonella enterica* serovar Typhimurium (3 – 5). Pathogenic, commensal, and probiotic strains of *E. coli*, however, frequently express the rdar morphotype semiconstitutively (5 – 9).

In this study, we sequenced the genomes of eight *E. coli* strains with rdar biofilm formation at 28°C and 37°C (Table 1). Recently isolated and minimally passaged strains include three commensal *E. coli* isolates from human feces (6) and four uropathogenic strains obtained from patients’ samples (reference 7 and this study). As a historical strain, we included ECOR 31 isolated from leopard feces from the *E. coli* reference strain collection (10).

**TABLE 1:** Strain information, sequencing, and genomic features of the eight *E. coli* draft genomes

Strain	Isolation source^a^	No. of sequence entities	Coverage (×)	*N*_50_ contig size (bp)^b^	Size (Mbp)^c^	G+C content (%)^c^	No. of genes (total/coding)^d^	No. of tRNAs^d^	Nucleotide accession no.
Tob1	Commensal human fecal isolate, Germany	4 polished contigs	68	NA	5.19	50.9	5,147/4,882	90	MIIH00000000
Fec67	Commensal human fecal isolate, Germany	97 scaffolds	173	411,704	5.20	50.6	5,115/4,918	79	MDRZ00000000
Fec101	Commensal human fecal isolate, Germany	100 scaffolds	192	418,989	4.97	50.7	4,942/4,751	79	MDYZ00000000
ECOR31	Commensal fecal isolate from leopard, USA	6 polished contigs	159	NA	5.44	50.6	5,328/5,095	90	MIIL00000000
No. 12	Human pyelonephritis isolate, Slovakia	112 scaffolds	125	349,216	5.10	50.5	5,009/4,807	78	MIIG00000000
B-11870	Human urosepsis isolate, Sweden	116 scaffolds	138	377,062	5.65	50.5	5,188/4,970	77	MIIJ00000000
80//6	Human UTI isolate, Estonia	90 scaffolds	172	367,346	4.98	50.6	4,886/4,697	79	MIII00000000
B-8638	Human urosepsis isolate, Sweden	140 scaffolds	170	190,633	5.21	50.7	5,143/4,917	77	MIIK00000000

a UTI, urinary tract infection.

b NA, not applicable.

c According to RAST server.

d According to NCBI Prokaryotic Genome Annotation Pipeline.

Genomic DNA from Tob1 and ECOR 31 was sequenced with the PacBio RS II system (Pacific Biosciences; NGI Uppsala, Science For Life Laboratory [SciLifeLab], Uppsala, Sweden). The assembly was done on SMRT portal version 2.3, using HGAP3 with default settings. The other strains were sequenced using an Illumina MiSeq version 3 platform with read length up to 2 × 300 bp (NGI Stockholm, SciLifeLab, Solna, Sweden). *De novo* assembly was performed using SPAdes (http://bioinf.spbau.ru/spades) (11). For calculations of coverage according to Lander and Waterman (12), see Table 1. Analysis by the Rapid Annotations using Subsystems Technology (RAST; version 2.0) server (http://rast.nmpdr.org/rast.cgi) (13–15) indicated a genome size between 4.97 and 5.65 Mbp and a G+C content between 50.5% and 50.9% (Table 1).

Contigs smaller than 500 bp were omitted, and the sequences were submitted to DDBJ/ENA/GenBank and annotated with the NCBI Prokaryotic Genome Annotation Pipeline. The number of genes is between 4,886 (*E. coli* 80//6) and 5,328 (ECOR 31) (Table 1).

Published genome sequences of *E. coli* isolates lack coupling to a distinct biofilm phenotype, but it is well known that even *E. coli* K-12 derivatives designated the same name derived from different laboratories can have different biofilm phenotypes (3, 16). The sequences of *E. coli* genomes with distinct biofilm phenotypes provide a profound basis to investigate the molecular mechanisms of semiconstitutive rdar morphotype expression.

### Accession number(s).

The whole-genome shotgun projects have been deposited at DDBJ/ENA/GenBank under the accession numbers listed in Table 1. The versions described in this paper are the first versions, respectively.
